# The Evolution of the Aseptic Method in Surgery

**Published:** 1898-07

**Authors:** Lawson Tait

**Affiliations:** F. R. C. S., Edinburgh and England; Professor of gynecology in Queen’s College, Birmingham; Surgeon to the Birmingham and Midland Hospital for Women, etc.


### Selection.

### THE EVOLUTION OF THE ASEPTIC METHOD IN SURGERY.

By LAWSON TAIT, F. R. C. S., Edinburgh and England,
Professor of gynecology in Queen's College, Birmingham ; Surgeon to the Birmingham and Midland Hospital for Women, etc.

I DESIRE to supplement the able paper of my friend Dr. Lewis S. McMurtry, of Louisville, in the February number of the *American Journal of Surgery and Gynecology*, because what I say must be largely of the nature of approval as to his results, something of the nature of correction and criticism and altogether the fulfilment of a desire similar to that by which he is clearly influenced, to reconcile discrepancies and remove difficulties. He looks at the shield ; so do I. But we don't look at the same side, and I think each side of value, though the values are different.

I begin with a difference : Dr. McMurtry is good enough to speak of " the Tait school of surgery " and to say that " many leaders of surgical opinion while practising asepsis derided all bacteriological discoveries and repudiated germ invasion as the cause of septic infection," and he in particular accuses me of this blunder. It would have been more convincing if he had quoted a single sentence from my own writings which would have established this charge, which is, indeed, absolutely the reverse of the truth. I have followed all these researches, and do so still, with the utmost diligence, and I welcome each fact with the utmost reverence. But what is a fact, is the question on which we so often split ! Look at the most recent researches on the bacteriology of the skin, surely a field most easily open to accurate investigation, and we see contradiction following assertion with bewildering certainty until we close our eyes in disgust. Look at the researches of sterilisation of the hands, a matter on which Dr. McMurtry most properly lays great emphasis, and we find the most violent discrepancies following one another as fast as sequent and inconsequent papers can be published. As in all new fields of research, far too great haste has been displayed and but half formed guesses have been shed broadcast as "established truths." That I have often mocked at and made fun of this kind of thing is true enough, but only when I found that from beginning to end it was false and incompatible with what I love to call "the science of the housekeeper."

That the air contains the germs of ordinary decomposition has been known ever since the days of cookery. That science revealed exactly what these germs were was a glorious victory, but it did not banish the great truths of the housekeeper. She knew that many things, such as moisture, temperature, specific gravity, absence of light and above all things, "life," modified or even prevented harm being done by the agents of decomposition, no matter what they were. She knew nothing led so certainly to the "tainting" of a piece of fresh meat as putting it on a dirty wet dish in a close atmosphere. To rub it dry, to cover it with powder well rubbed in (if an antiseptic like pepper all the better), but most of all, to place in a current of dry, cool air, was to keep it fresh perhaps for weeks. She knew in fact how to encourage her materials to resist the germs of putrefaction until she finally made them entirely aseptic by cooking them in a tin can and soldering them up. There they kept for years. But she also knew that the simplest forms of life could resist those causes of decomposition so long as life remained. She knew that nonfertilised eggs will keep only a very short time, but that fertilised eggs will, under proper conditions, keep three weeks or more. She knew in fact that this mysterious thing, "life," is the most perfect antiseptic we have, and this prime fact is that which makes all "cultures" and all laboratory experiments fail absolutely in giving results which can with safety be applied to the living being, particularly to man.

When we come to consider man we find all our facts at sixes and sevens and all theories of no avail. The laboratory fails utterly; and whilst in the processes of his body we may see veri-similitude with those of the lower animals, we are met at once by facts which show they are not. Bennett and Rutherford made hundreds of experiments in making biliary fistulæ in dogs and they had an enormous primary mortality, whilst the same operation in the human being has practically no mortality at all. If successful in the dog to the purport of diverting all his bile into test tubes, the dog dies inevitably of starvation. If the same result is obtained in the human subject, he or she fattens on the loss of the bile and suffers in no way from it save in the trouble of skin excoriation. The common femoral artery of a large dog will be closed by four hours' temporary metallic compression, whilst forty hours of the same pressure cannot be depended upon to close a human artery of the same size. Not only this, but human beings alive differ in their individual results from exactly similar conditions in ways altogether irreconcilable with the laboratory facts of bacteriology.

No disease is so certainly recognised in all its detail as typhus fever, for in it alone we have a disease of which not only the clinical facts are absolutely known, but its causation has been established with mathematical certainty. We knew, long before the days of bacteriology, that it arose from and was spread by a living thing. Whether that living thing has been spotted and named I do not yet know and it is wholly immaterial.[1] But Sir Henry Littlejohn has proved that it is a thing, let us say a living organism, which can be created or brought into existence at will, and at will destroyed. It is, therefore, probable, as Darwin suggested, a living organism of some very ordinary kind, which becomes possessed of its terrible powers simply by living on the overcrowdings of human beings. These powers are possibly derived from its living on the albuminoid substance condensed on walls or other cold surface from the rebreathed air of humanity. There is no other possible explanation of such experiments conducted in the Black Hole of Calcutta and in the slums of Edinburgh by Sir Henry Littlejohn and Sir William Chambers. Another stage of the argument is this, that whilst the germs of decomposition will attack every dish of dead milk alike in the same dirty and badly ventilated dairy, the germs of typhus will not attack every person living in the same dirty, overcrowded and ill-ventilated house in the West Port; nor of those attacked will the result be at all the same in any two cases. Some will escape with slight headache, whilst others will die in forty-eight hours of a hemorrhagic attack, before even the rash is developed.

This lands us at the difference between the asepticists and the antisepticists: the former wanted the nidus of the germ altered so that neither pabulum nor resting-places for it could be found; the latter fled after the spores with lotion and plasters and sprays and irrigations, leaving the dirt alone and the overcrowding! Let us try the history of facts.

1. I think we may take it from the contradictory evidence recorded by a learned writer in the *British Medical Journal*, April 2, 1898, that it has not yet been identified.

Lister and I started from common ground. He entered the Edinburgh infirmary as assistant surgeon in 1857 and I as a student in 1860. Syme was professor of clinical surgery and had a special building of his own, lighted and ventilated on both sides, two stories in height and with a reasonable number of beds, the exact number I do not remember, but I should say about thirty. At least the wards always had a startling contrast in every way with those in the contiguous building in matters I shall refer to in detail, and yet the two establishments were separated only by two valves of a swing door.

Personally Syme was the personification of the best type of gentleman, always perfectly dressed in his old-fashioned way, and as clean as a new pin. From his boots to the top of his head no one ever saw dirt, disorder, or the appearance of hurry. He was always washing his hands; I think I may say he washed them every time he touched a patient. His assistants had to be like him, and his old-fashioned nurses were noted for their tidiness and cleanliness everywhere. Through the folding doors what a difference! Everywhere hurry, untidiness, and no mark of what I have just praised in Syme. There was a good deal of dandiness, but much dirt; I shall not venture on any detailed description, though all who took part in it as principals have gone to the majority, yet I might traverse into unintentional exaggeration, and I might hurt the feeling of some who took part in the affairs of the old Edinburgh Infirmary after my time. Suffice it to say, it was a four-story building, badly lighted, very badly ventilated, crowded with students for many hours of the day, with a very insufficient number of nurses, who were void of training; that the wards stank, that they were overcrowded and marked by numerous faults in cleanliness and hygiene which would not be tolerated now in the oldest-fashioned institution in the country. There is an authentic word picture of a similar state of affairs at the same time which will serve to show what Simpson meant by "hospitalism." I shall quote it at length: "The operating table was of wood and of fabulous age. It was sometimes thought necessary to have a new mattress for this table when the stuffing was found to be matted together in lumps by the blood which had during many years soaked through its covering. The only correct garb of the surgeon was a frock coat (the oldest and shabbiest in his wardrobe), which was kept in the surgeon's room and never renewed or cleaned during his twenty years of operative work." (Syme always turned up the sleeves of a dress coat in which he might have shown himself before the operation before his Queen at Windsor.) "The operating theatre attendant was permitted to employ his spare time in the post mortem room, the surgeons came straight from the dissecting room to operate after simply washing their hauds; ligatures were used which had been already spoiled by handling with blood-stained fingers to sew up wounds on a second case." And at Edinburgh these ligatures were always worn ostentatiously by the house surgeon, like a badge of knighthood, in the buttonhole of a coat which often rivaled that of his chief for dirt and evil condition. What were the causes of the extraordinary differences separated by the memorable swing doors?

The prime and real causes were, first of all, that Mr. Syme was a master, that he held his appointment for life, was a rich man and he made his underlings do as he wished. Besides, he cared a great deal for results, as was shown in the notorious trials about perineal section. He was not merely desirous of doing an operation, but he was desirous of seeing the patient cured and well years afterward. Further, he was a great surgeon, in every sense the greatest I have ever seen. Nothing charmed him more than to show us a man upon whom he had done some tremendous bit of work years before, and this alone can be the object of true surgery. On the other side of the door the men were on short tenure of office and,, necessarily, some of them were quite incompetent for it. They dashed at their work, they hurried it, they crowded all they could into the ten years they had of it. I saw some awful things in my six years of pupilage in the old Edinburgh infirmary, things surgeons could not possibly bring about now if they tried. It is now a very parrot cry that operations are done now which never could have been done, never were attempted before the days of Lister. This is absolute nonsense if it is meant that Lister has made surgery any safer, and the evidence is at the hands of Lister's own father-in-law, Syme himself. It was etiquette for the physician or surgeon responsible for the treatment of a fatal case to appear at the post mortem. Syme seldom appeared, not that he would not come, for no one ever displayed, to my mind, a keener anxiety to know why he had failed. But look at his gigantic enterprises, enterprises which often, in my own recollection, brought Lister from Glasgow and Bickersteth from Liverpool to have the privilege of assisting. Does Lord Lister remember as I do the awful inguinal aneurism in which the patient was slit, as it seemed, from the middle of the thigh up to the umbilicus? the gluteal aneurisms, the removal of the whole lower jaw, the fearful esophagotomies and, most of all, the thirty-seven consecutive cases of ligation of the femoral artery for popliteal aneurism, not one of which died. Perhaps, most noteworthy of all, though least capable of theatrical display, were his numerous perineal sections, and all this without a single antiseptic, but with a detailed care for asepsis which was perhaps as great as my own. He would have no assistants' fingers in wounds he made, fingers whose dirt he had no means of gauging and no means of depriving of this deadly influence. Let us remember that Keith, who at least led us into the diminished mortality of abdominal surgery, was Syme's patient (first), then pupil, house surgeon and assistant. Had it not been for the unfortunate quarrel with Lizars in early life, Syme would never have been prejudiced against abdominal surgery. He would have established ovariotomy in the "thirties," and all that has since flown from it would have been done by him, and neither the schools of Lister nor that of Tait would ever have been heard of. Let us hear no more of the nonsense about the bad surgery of pre-Listerian times as having been cured by Lister.

Let me now take Dr. McMurtry somewhat in detail, historically, from the year 1867, that in which I was permitted to pursue an independent course in life and in surgery. As I have said in public more than once, I left Edinburgh with at least one conclusion determined in my mind, that I should never deliberately open the abdomen. The results I had seen in Edinburgh were truly awful—some thirty cases and not a recovery! But then they were all on the other side of the swing door! Fortunately for me, Simpson's early papers on "hospitalism" appeared, and the "battle of the sites" began, at first a sort of duel between Simpson and Syme—to be expected as a matter of course. At first Syme saw no reason for a change of site for the Edinburgh Infirmary, and small blame to him, when considered only from his position. But he suddenly changed his attitude—we have never been told why, but the guess is that it was the difference in the other side of the door which converted him, when he had made known to him its awful significance.

Then he battled only for an alternative site. In 1868 I broke through my formed resolutions and of my first ten ovariotomies only one died, the mortality of an untrained beginner, but the mortality maintained by the intra-peritoneal method unaided by drainage and purgation. Then evil ways fell upon me and I went on with the clamp, raising my mortality to nearly 50 per cent. 1878 saw the short ligatures re-introduced by Bantock and myself, and the principle of perfect segregation of my patients, together with the free use of drainage by the tube and the Seidlitz powder, and the mortality fell to 5.5 per cent. through the long series of 1,350 cases, published in the report of the Birmingham Hospital for Women for 1893, all hospital cases with report of the every essential detail authenticated by the managing committee of the hospital and my colleague, Dr. Savage, and myself, at whose hands the actual work was done in very nearly equal shares. In my hands were no Listerism, no chemical antiseptics, nothing but plenty of soap and water vigorously applied, and strict attention to detail. In Dr. Savage's work, as he permits me to say, he followed Lister in fashion of his own, varying from time to time, and the fact that we arrived at results almost identical is more convincing than anything else that chemical antisepsis was an altogether unnecessary factor. No malicious assertions that our statistics have been cooked can touch such a solid fact as this, and our critics have been invited to go over the record for themselves.

Dr. McMurtry says, what I have over and over again asserted, that "the great facility of absorption possessed by the peritoneum made abdominal surgery the field of conflict for deciding the comparative merits of asepsis, and the relation of bacteria to wound infection generally." Here another triumph was secured by Mr. Sampson Gamgee, whose system of dry-dressing by absorbent and expanding cotton wool constitutes one of the most brilliant contributions to modern surgery, from which I have made my departure for sixteen years. Equally true is what Dr. McMurtry says of animal ligatures, but then I limit this to fine silk, the best, the least dangerous and most trustworthy of all ligatures, and for every purpose.

"Hospitalism" and "the battle of the sites" have transformed all the hospitals from shambles into palaces. Money flows in and honors follow it, so soon as the rebuilding and reconstruction of an old hospital is proposed. Small hospitals under various names are spread over this land in countless numbers, and with these more complete segregation of the patients is the chief object. And when a due interval has elapsed anyone who collects a few thousands of their cases of amputation and contrasts the result with the terrible record of my "hospital mortality" for fifteen years, 1860 to 1875, will prove to the world the immense change for the better by Simpson's agitation.

The case against Listerism is very tersely put by Dr. McMurtry in these words: "Much confusion arose from the failure to recognise the power of the tissues to resist germ invasion and that in all surgical operations a certain degree of bacterial infection is overcome by this resistance." A corollary to this is, of course, that the extent of the wound aided by the increase the extent of bacterial infection. Had it not been so, Lister, to be consistent and careful of his own welfare, ought to have been shaved every morning under the spray, or at least under an irrigation, for one germ would be as fatal as a million. Again, Dr. McMurtry's words are true to the letter when he says: "Reliance upon antisepsis begot a disregard for mechanical cleanliness and established a confidence in chemical germicides to atone for slovenly methods throughout an operation." So we heard men in great surgical positions say in society debates that they would go unhesitatingly to the operating theater from the post mortem room or the dissecting table and perform an ovariotomy, provided they were allowed to wash their hands in a 5 per cent. solution of carbolic acid! This was our danger, but there was another as great, perhaps greater: Listerism became a fetish, a royal road to surgical success, by which men ventured to travel in surgery whose hands fitted them only for field labors.

So far Dr. McMurtry and I are agreed, but here we take different paths. He says that our discussions have now ceased and that throughout all civilised nations, where surgery is taught and practised as a science, the principles of Lister are accepted and applied. It is not so; it shall not be so as long as I live and can write. The agent may be the germ of decomposition or a specially and sportively-endowed germ, but the answer to him is the condition of his surroundings. With exact and scientific recognition of the germ seems to me only the beginning of the mystery. I believe, with Keith, as quoted by McMurtry: "It was the willing and tender, though unclean hand of the surgeon, that carried the poison into the wounds." As my experience grows, as I ponder year after year over fatal cases, this conviction gets stronger and stronger.

I have eliminated every cause of death (save accident, and that so far as I can), save the poisoning hand, and there every now and then I am beaten, beaten in the most sudden and overwhelming fashion, in cases where there can be no other explanation, and in some where I have seen it plain enough. Thus I operate on a huge perineal abscess, and the smell is enough to empty the house. My fingers are soiled by the horrible pus, and I wash and wash with germicides and perfumes and the smell remains for days. Nobody will persuade me that it is the presence of germs that continues the awful smell. It is the presence of something far more potent than germs, probably a secretion of theirs, which soaks deep, deep into the skin, far beyond where a germ could go. The slightest access of these germs to an abraded finger means something little short of death. A post mortem was held on one of my patients with such an abscess as this round the kidney, and of the three men present two, who had no contact at all actually with the putrid pus, died of putrid sore throat in a few days, and the third was seriously ill. This peculiar putridity is common in perineal abscesses and in abscesses of the cheek. It is also met with in and around the kidney, in the peritoneum when the bladder is an active factor in the suppuration, and in all cases of purulent peritonitis—and chiefly of puerperal origin. It is a most deadly thing when introduced into the peritoneal cavity by the finger of the surgeon, even days after his contact with it. Some years ago I opened the abdomen of a woman four days after her labor for purulent peritonitis and encountered this fearful putridity. Having strong suspicions of its deadly character, I soaked my hands for hours in various germicidal lotions as hot as I could bear them, till my hands were like those of a washerwoman. On the third and fourth days after, I operated on two simple ovarian tumor cases, and the rapidity with which these two women succumbed was altogether shocking, the post mortem examination leaving no other guess than "acute septicemia." A perfectly parallel experience has been the melancholy fate of one of my recent assistants.

Then, when I find the outcome of the most recent investigations of the scientists, I think of Keith, conspicuously devoid of any science, and I conclude that he knew more and was humbler in his belief than the proud-hearted investigators. I am on his side. Even if every word of the ever-changing doctrines and details of the Listerian system were true (and as I have often said there is much true, but not much new), I say it is only another notch in the tree, and not a big one. Even if it were true what Dr. McMurtry claims for it, now we see what a humiliation it would be to have to confess that after all this fight for a quarter of a century it results only in the old advice to the Assyrian, "Go, wash, and be clean."

If we had watched Syme more closely, and had listened to Simpson with ear other than that of the adder, closed and deaf, we had not needed this fight.—*Am. Jour. Surg. and Gynecology*, May.

---

CONTRACT medical practice was recently discountenanced by resolution by the Connecticut State Medical Society. It was held that the making of contracts for medical service at a fixed annual rate with lodges, mutual benefit societies and medical clubs was unprofessional.

